# Public Knowledge and Attitude towards Epilepsy and Its Associated Factors: Community-Based Cross-Sectional Study, Ethiopia, 2019

**DOI:** 10.1155/2020/6801979

**Published:** 2020-07-17

**Authors:** Abate Dargie Wubetu, Elyas Admasu Basha, Nigus Alemnew Engidaw

**Affiliations:** Department of Psychiatry, Debre Berhan University, Debre Berhan, P.O. Box: 445, Ethiopia

## Abstract

**Background:**

Epilepsy is a disease of the brain defined by any of the following conditions. First, at least two unprovoked (or reflex) seizures occurring greater than 24 hours apart, and secondly, the presence of one unprovoked (or reflex) seizure and a probability of further seizures. Due to persisted misunderstandings and negative attitudes, individuals living with epilepsy live with a poor quality of life. Therefore, this study aimed to assess the community general knowledge about epilepsy and attitude towards person living with epilepsy and its associated factors.

**Methods:**

A community-based cross-sectional study conducted from March 10 to June 10, 2019, to assess the community general knowledge and attitude towards epilepsy and its associated factors. Data were entered into Epi data version 3.1 and transported to SPSS version 21 for analysis.

**Results:**

596 study participants participated in a response rate of 98%. Among the study participants, 43.6% (95% CI: 39.6, 47.5) had poor knowledge and 41.3% (95% CI: 37.4, 45.1) had an unfavorable attitude. Lack of modern education, married, never witnessed a seizure, and not hearing the term epilepsy showed statistically significant association with poor knowledge about epilepsy. Lack of modern education, earning less than 1000 Ethiopian birr, not witnessing seizure, not hearing the term epilepsy, and half to one-hour walking time from health facility variables showed statistically significant association with the unfavorable attitude about epilepsy.

**Conclusions:**

The current study revealed that nearly half of Debre Berhan dwellers have deficits in terms of general knowledge about epilepsy and attitude towards a person living with epilepsy. Poor knowledge about epilepsy and unfavorable attitude towards a person living with epilepsy are likely to have an important impact on stigmatization and treatment-seeking behavior, and it should be given due attention. It would be better if health educators give special emphasis for the individuals with predictors of poor knowledge and unfavorable attitude.

## 1. Background

Epilepsy is a disease of the brain defined by any of the following conditions. First, at least two unprovoked (or reflex) seizures occurring greater than 24 hours apart, and secondly, the presence of one unprovoked (or reflex) seizure and a probability of further seizures similar to [1].

There are more than 70 million people living with epilepsy in the world; of them, nearly 80% live in developing countries. Epilepsy remains a major health problem in developing countries. The burden of epilepsy is not only because of its health implications but also because of its socioeconomic and psychological effects [2, 3]. Half of the epilepsy cases live in developing countries. The highest levels of untimely death due to epilepsy are found in these countries [4, 5]. The 12-month incidence and prevalence of epilepsy in sub-Saharan Africa is high [6]. The incidence of epilepsy in Ethiopia is 64 per 100,000. The age-specific incidence was highest in the group aged 0–9 years [7]. The prevalence of epilepsy in developing countries varies from 0.5 to 1 percent. It is more prevalent in rural communities [8, 9].

The knowledge, attitude, and practice of the community about epilepsy are poor, even among people who know individuals living with epilepsy. Lack of knowledge about the causes of epilepsy has been associated with unfavorable attitudes, poor awareness, and stigma [3, 8, 10]. Lack of understanding about epilepsy is a leading cause of discrimination in the community [11]. Most studies showed that a high number of people heard about epilepsy. However, most of them had a lower level of knowledge. A significant number of people considered that epilepsy is a mental disease, hereditary disease, contagious, evil spirit, or god's curse [12, 13].

Due to the unfavorable attitude towards a person with epilepsy, many people do not want to work and live a person living with epilepsy. Large numbers of people do not want even to shake hands with epileptic individuals. Individuals with an unfavorable attitude try to keep their children away from these patients. A person living with epilepsy may also have perceived stigma [14]. Unfavorable attitude and poor knowledge continue to root a negative influence on the management of epilepsy in African countries. The disorder leads to irrational belief, discrimination, and stigma in many of African countries. Spiritual and sociocultural dogmas influence the type of treatment received by individuals with epilepsy and epilepsy in the most reported reason for school rejection as it seen contagious and shameful [15, 16].

A compiled data of articles across the world showed that the magnitude of poor knowledge about epilepsy ranges from 40% to 86% [17–21]. Unfavorable attitude towards patients living with epilepsy also ranges from 28% to 87% [11, 19–24].

In the last decade, there has been an increase in literacy rates and increased access to technology in the Debre Berhan population (the study area). However, it is uncertain to decide the increased literacy rate had a positive effect on knowledge about epilepsy and attitude toward persons living with epilepsy. Poor awareness about epilepsy and unfavorable attitude towards patients living with epilepsy are sources of stigma and discrimination and are predictors of poor quality of life and delay for modern treatment in people living with epilepsy. Studying knowledge about epilepsy and attitude towards persons living with epilepsy in Debre Berhan town enables us to know the level of poor knowledge and unfavorable attitude and enables us to identify the factors that result in poor knowledge and unfavorable attitude. Findings could benefit the policymakers, district health team, community members, affected families, and people with epilepsy in designing strategies. Furthermore, the study possibly generates information in the area of the topic for large-scale researchers to investigate further empirical evidence to control those factors attributable to poor knowledge and unfavorable attitude towards epilepsy in the study area.

## 2. Methods

### 2.1. Study Period and Area

The study was conducted from March 10 to June 10, 2019. Debre Berhan is the largest town of North Shewa of the Amhara region and is located 130 km northeast of Addis Ababa. It comprises a total population of 108,825 out of those 49,208 were males and 59,617 were females within nine kebeles.

Debre Berhan town is located in North Shoa Administrative Zone, Amhara National Regional State, Ethiopia. The town has nine administrative kebeles. In the town, there is one public referral hospital and one private general hospital, three health centers, nine health posts, and five private specialty clinics, which render health services to the community.

### 2.2. Study Design, Population, and Variables

The community-based cross-sectional study design was conducted to assess public general knowledge and attitude about epilepsy and its associated factors. The source population was all households in the study area. All households found in the selected kebeles (subcities) considered as study population. Each individual in the selected household in which the actual data collected was considered as the study unit. All individuals who were living permanently (at least six months), aged 18 years and above, were included in the study. The following variables were considered as dependent and independent variables of this study.

Dependent variables: knowledge towards epilepsy (poor/good) and attitude towards epilepsy (unfavorable/favorable).

Independent variables: sociodemographic factors: age, sex, religion, marital status, address, academic status, residence, family role, and ethnicity.

Health facility factor: availability and distance.

Experience with epilepsy: having a family member with epilepsy, having epileptic friend, and witnessed a seizure.

Information factors: magazines, TV, radios, family members, healthcare provider, and training

### 2.3. Sample Size Determination

A single population proportion formula was used to calculate the sample size of the study by assuming 4% margin of error, 95% confidence level, and 10% nonresponse rate. And, the population proportion of unfavorable community attitudes in the related study in Ethiopia was 64.4% [21]. The result of the sample size became 551 participants. Considering 10% (55 participants) of the nonresponse rate, the final sample size of the study was 606 individuals.

### 2.4. Sampling Technique and Procedure

The authors had selected two subcities (kebeles), out of the total nine kebeles by the lottery method. A systematic random sampling method was used to select households for the study from the selected kebeles. The sampling fraction was eight, and our starting household was selected by using the lottery method from the first eight households. For those households, which have more than one eligible participant, one study unit was selected by the lottery method.

### 2.5. Data Collection and Tools

The data of the study were collected by using pretested 10- and 12-item interviewer-administered questionnaires for knowledge and attitude, respectively. The questionnaire was translated into local languages (Amharic) and converted into English by language experts to ensure consistency. The questionnaire was pretested before two weeks of the actual data collection period among 10% of the sample size (*n* = 60). The questionnaire was pretested among community members who did not study participants. The pretested data were not included in the final data analysis. The pretested questionnaire was modified based on the result of the pretest. The reliability of the pretested questionnaire was checked by using Cronbach's alpha test, and its value was 0.90 for attitude and 0.81 for the knowledge-screening tool. Five undergraduate public health graduates conducted data collection. The supervisors were two masters' mental health professionals. The interview was performed at the study participants' household, and the issue of privacy was maintained.

#### 2.5.1. Attitude

The section on attitude had 12 items and 4-point Likert scale response options consisting of “strongly agree, agree, disagree, and strongly disagree.” The response options were given (scored) a point of 4, 3, 2, and 1, respectively. The minimum score is 12, and the maximum score is 48. Individuals who scored below the mean (*X* = 34) and ≥ mean (*X* = 34) were categorized as unfavorable and favorable attitudes, respectively.

#### 2.5.2. Knowledge

The general knowledge of the respondents about epilepsy was assessed by using 10-item pretested questionnaire, which has yes/no choices. For each true answer to the questions on knowledge, one point is given and zero points given for wrong answers. The total score ranges from zero to ten. The mean of the score was used to categorize the individuals' knowledge about epilepsy. Those who scored 7–10 points were considered as having good knowledge and those with 0–6 points had poor knowledge.

### 2.6. Data Quality Control

The questioner translated from English to Amharic and back to English to check the consistency. To ensure the quality, the questionnaire checked for completeness, accuracy, clarity, and consistency by the principal investigator. To maintain the quality of the data the questionnaire pretested among 10% (60 study participants) of the study households, the pretested data were not included in data analysis. Intensive training and close supervision were held during the whole data collection period by the research advisor.

### 2.7. Ethical Consideration

Ethical clearance was obtained from the ethical review board of Debre Berhan University's Research Ethics Committee. The study participants were informed about confidentiality, and oral consent was obtained from each participant.

### 2.8. Data Processing and Analysis

After the data checked for completeness and consistencies, the questionnaires coded and entered using Epi-data version 3.1 and exported to SPSS version 21 for analysis. Descriptive statistics was presented by using texts and tables. Both bivariate and multivariate binary logistic regressions were conducted to identify the associated factors with the outcome variables. Variables with a *p* value of less than 0.2 during bivariate analysis were exported to multivariate analysis to adjust the effect. During multivariable analysis, variables with a *p* value ≤0.05 and odds ratio with their 95% confidence interval were considered as significantly associated factors with the outcome variables. Assumption tests and model goodness of fit were checked.

## 3. Results

### 3.1. Sociodemographic Characteristics of the Dwellers

Six-hundred six (606) households selected for the sample, and 596 were successfully interviewed, yielding a response rate of 98%. Nearly half of the study participants, 264 (44.3%), were females and 332 (55.7%) were males. The median age of the respondents was 30 years, and the interquartile range was 15 years. Among the 596 study participants, the majority (470 (78.9%)) were orthodox Christian. The remaining, 81 (13.6%) participants, were Muslim and 45 (7.6%) were protestant in religion. The majority of the respondents, 494 (82.9%), were Amhara by ethnicity followed by Oromo which accounts for 67 (11.2%). Regarding the income of the respondents near to half, 270 (45.3%) had a monthly income of >3000 followed by those whose monthly income is between 1000 and 2999 and <1000 which accounts 243 (40.8%) and 83 (13.9%), respectively. Among the total participants, more than half 333 (55.9%) were married, whereas the other 263 (44.1%) were single. Majority, 445 (74.7%), of the respondents had a family size 1–3 followed by those whose family members were 4-5 (136 (22.8%)) in number (Table 1).

### 3.2. Information, Experience, and Health Facility-Related Factors of the Dwellers

The majority of the respondents (546 (91.6%)) had heard of epilepsy as a disorder. The commonest sources of information about epilepsy were from their family or friends (305 (55.9%)), while the rest were through radio/magazines, television/training, and health professionals, which account 92 (16.8%), 85 (15.6%), and 64 (11.7%), respectively. Majority (475 (79.7%)) of the respondents had witnessed a seizure in the past but only 151 (25.3%) and 46 (7.7%) had a friend or a family member with epilepsy, respectively. Among our study participants, all 596 (100%) have health facilities near to their home, and the majority (483 (81%)) takes less than 30 minutes, whereas 113 (19%) takes 31–60 minutes.

### 3.3. Magnitude of Poor Knowledge among the Dwellers

Among our study participants, near to half (43.6 (95% CI: 39.6, 47.5)) had poor knowledge about epilepsy. This study shows that 192 (32.2%) of the study participants reported seizure attacks did not originate from the brain. 278 (46.6%), believe the cause of epilepsy is an evil spirit, and 310 (52%) reported infection or injury cannot cause a seizure disorder. Among the study participants 161 (27%), 253 (42.3%), and 236 (39.5%) of the respondents said epilepsy is contagious, and traditional medicine is best for epilepsy treatment and would not mind their child to play with an epileptic child. Among the study participants, 101 (17%) thought that all children who experience convulsion have a seizure disorder. One-hundred seventy-seven (29.7%) thought seizure disorders occur in the family as well, and 176 (29.5%) did not know about the presence of different types of seizure disorders. 203 (34.1%) thought there is no medical test for diagnosing seizure disorder, and 216 (36.2%) reported the seizure disorder can be treated with antiepileptic drugs.

### 3.4. Magnitude of an Unfavorable Attitude of the Dwellers

In this study, 596 participants, near to half (41.3 (95% CI: 37.4, 45.1)), had an unfavorable attitude towards epilepsy. The number of study participants who did not agree about their family members marrying a person living with epilepsy accounts for 126 (21.1%). Moreover, 105 (17.6%) did not agree to recruit person with epilepsy, and 95 (15.9%) kept their child away from contacting epileptics. Almost fifteen percent, 91 (15.3%), did not agree to live together with person with epilepsy (Table 2).

### 3.5. Associated Factors of Poor Knowledge

To determine the factors associated with the knowledge about epilepsy, binary logistic regression was done and computed with each independent variable. In binary logistic regression, respondent's age, marital status, educational status, occupation, income, family role, witnessed a seizure, and heard the term epilepsy in the past were the variables that showed statistically significant association with the outcome variable that is knowledge about epilepsy. In multivariate analysis, educational status, marital status, witnessed a seizure, and heard the term epilepsy in the past were the variables that showed statistically significant association with the outcome variable that is knowledge about epilepsy. Respondents who did not attend modern education and those who completed secondary education were around four times (AOR = 3.74, 95% CI: 1.80, 7.70) and two times (AOR = 2.13, 95% CI: 1.27, 3.59) more likely to have poor knowledge than those who completed grade 12, respectively. Those participants who were married were two times were more likely to have poor knowledge (AOR = 1.88, 95% CI: 1.20, 3.21) than those who were single. Those who did not witness seizure were two times more likely to have poor knowledge (AOR = 1.79, 95% CI: 1.10, 2.90) than those who witnessed seizure in the past. A respondent who had not heard the term epilepsy through different mass media or training was around three times more likely to have poor knowledge (AOR = 2.67 95% CI: 1.27, 5.59) than those who had heard about epilepsy (Table 3).

### 3.6. Associated Factors of Unfavorable Attitude

The following factors were found to be significantly associated with the attitude towards epilepsy in bivariate analysis: respondent's sex, age, monthly income, educational status, marital status, family role, occupation, whether they had witnessed seizure, whether they had a family member with epilepsy, whether they had a friend with epilepsy, whether they had heard about epilepsy, and distance of their home from a health institution. In multivariate analysis, educational status, average monthly income, walking time from home to the health institution, witnessed a seizure, and heard the term epilepsy in the past were the variables that showed statistically significant association with knowledge about epilepsy.

Respondents who earn less than 1000 Ethiopian birr (ETB) were 2.5 times higher in odds to have unfavorable attitudes than those respondents who earn an average monthly income greater than 3000 (AOR = 2.52, 95% CI: 1.34, 4.7). Those respondents who were not attending modern education were 2 times more likely to have an unfavorable attitude (AOR = 2.1, 95% CI: 1.10, 4.44) than those who completed grade 12.

There was also a significant association between level of attitude and whether the respondent has witnessed seizure or not; those respondents who had not witnessed seizure were two times likely to have unfavorable attitude than those who had witnessed a seizure (AOR = 2.27, 95% CI: 1.35, 3.80). A respondent who did not hear the term epilepsy through different mass media or training were five times more likely to have unfavorable attitude than those who had heard information about epilepsy (AOR = 4.73 95% CI: 2.04, 11.00). Further, those respondents who lived far from health institutions (>30 minutes on foot walk) were two times more likely to have unfavorable attitudes than respondents who lived near to health institutions (AOR = 1.77, 95% CI: 1.06, 2.79) (Table 4).

## 4. Discussion

According to the result of this study, 43.6% (95% CI: 39.6, 47.5) of the respondents had poor general knowledge and 41.3% (95% CI: 37.4, 45.1) had an unfavorable attitude towards epilepsy. Those who had not attended modern education, attended secondary education, were married, never witnessed a seizure, and did not hear the term epilepsy showed statistically significant association with poor knowledge about epilepsy. The level of education, earning <1000 Ethiopian birr, did not attend modern education, did not witness a seizure, did not heard the term epilepsy, and half to one hour walking time from health facility showed statistically significant association with an unfavorable attitude.

The magnitude of poor knowledge was in line with other studies conducted in Ethiopia [18, 21]. But, it is much lower than studies done in southwest Ethiopia [19] and the United States [17]. This might be due to the study year. The study from the United States was conducted in 2003, and the study conducted in Ethiopia was during 2015.

Regarding the attitude towards epilepsy in our study, 41.3 (95% CI: 37.4, 45.1) of the respondents had an unfavorable attitude towards a person with epilepsy. This study result is in line with the study conducted in South India [11]. But, this study finding is lower than that of the study conducted in Ethiopia [19, 21], Uganda [22], Nigeria [23], and Pakistan [24]. This might be a difference in community belief and literacy rate across the study areas and countries.

The current study result is higher than that of the study conducted in South India [11]. This might be because, in this study area, more than half of the respondents had an experience with epilepsy. Therefore, those individuals may have a favorable attitude. Negative attitudes were reinforced by beliefs that epilepsy is a kind of insanity.

Respondents who did not attend modern education were around 4 times more likely to have poor knowledge than those who completed grade twelve. This finding is in line with the study conducted in Ethiopia, the United States, and Ghana [11, 21, 25]. The possible justification might be individuals in higher education may exposed to different courses about epilepsy. In fact, it is expected to have a knowledge change while academic status is increased. The result reveals respondents who had not heard the term epilepsy through different mass media or training were around 3 times more likely to have poor knowledge than those who had heard information about epilepsy. This finding is in line with the study conducted in Ethiopia [21]. Hearing the term epilepsy may invite individuals to ask/read about epilepsy and this allows having good knowledge.

This study shows that those respondents who did not hear the term epilepsy through different mass media or training were 5 times more likely to have unfavorable attitude than those who had heard information about epilepsy. This association is in line with the study conducted in Ethiopia, which shows respondents who had heard information about epilepsy had favorable attitude compared with those who had not had information about epilepsy [21]. This might be because mass media and pieces of training are the main strategies to change people's attitudes towards persons with epilepsy.

Study participants who earn less than 1000 Ethiopian birr were more likely to have unfavorable attitude than who earn greater than 3000 Ethiopian birr per month. The possible reason might be because having more monthly income is associated with higher in participants' educational level. Living far from the health institution was also a contributor to having an unfavorable attitude, and this might be due to decreased exposure level for health issues like epilepsy.

### 4.1. Strengths and Limitations

Since the data were collected by the interviewer-administered technique, there may be a problem with an estimation of the dependent variables. Clarifications were given to the participants about their fear of correctness of the responses. The clarification about the participants' best responses was required. This study is a cross-sectional study, and therefore the associations between variables described may not necessarily be causal or explain the change of knowledge and attitude over time in the source population.

## 5. Conclusions

The current study revealed that nearly half of Debre Berhan dwellers have deficits in terms of general knowledge about epilepsy and attitude towards person living with epilepsy. Poor knowledge about epilepsy and unfavorable attitude towards a person living with epilepsy are likely to have an important impact on stigmatization and treatment-seeking behavior, and it should be given due attention. It would be better if health educators give special emphasis for the individuals with predictors of poor knowledge and unfavorable attitude.

### 5.1. Recommendations

It is advisable for an education program aimed at improving knowledge and attitude towards epilepsy held in the study area. It will be better to use an opportunity to give health education at both inpatient and outpatient treatment units. The care provider should give health information for the client and attendants that promote knowledge and attitude towards epilepsy during the health care visits.

## Figures and Tables

**Table 1:** Sociodemographic characteristics of the study participants in Debre Berhan, 2019.

Variables		Frequency	Percent
Age in years	18–35	372	62.4
	35–45	130	21.8
	>45	94	15.8

Occupation	Farmer or housewife	98	16.4
	Private workers	170	28.5
	Daily laborer	61	10.2
	Student	65	10.9
	Government employee	202	33.9

Educational status	Had not attended modern education	116	19.5
	Primary	95	15.9
	Secondary and pre.	145	24.3
	>12 grade	240	40.3

Family role	Family head	386	64.8
	Son/daughter	91	15.3
	Living alone	119	20

**Table 2:** Attitude towards people living with epilepsy in Debre Berhan, 2019.

Attitude towards epilepsy	Strongly agree (%)	Agree (%)	Disagree (%)	Strongly disagree (%)
Agree to work with epileptics	141 (23.7)	289 (48.5)	103 (17.3)	63 (10.6)
Agree to have close relation with epileptics	129 (21.6)	271 (45.5)	127 (21.3)	69 (11.6
Agree to live together with epileptics	114 (19.1)	251 (42.1)	140 (23.5)	91 (15.3)
Epileptics should not be isolated	280 (47)	244 (40.9)	53 (8.9)	19 (3.2)
Epileptics can manage their family	142 (23.8)	304 (51)	100 (16.8)	50 (8.4)
Agree to shake hands of epileptics	251 (42.1)	274 (46)	36 (6)	35 (5.9)
Keep child from contacting epileptics	120 (20.1)	244 (40.9)	137 (23)	95 (15.9)
Agree to recruit epileptics as a servant	74 (12.4)	182 (30.4)	235 (39.4)	105 (17.6)
Agree with family member marry epileptics	72 (12.1)	177 (29.7)	221 (37.1)	126 (21.1)
Epilepsy is treatable disease	168 (28.2)	290 (48.7)	84 (14.1)	54 (9.1)
Epileptics should not learn in schools	259 (43.5)	195 (32.7)	77 (12.9)	65 (10.9)
Epileptics can lead a healthy lifestyle	154 (25.8)	273 (45.8)	125 (21)	44 (7.4)

**Table 3:** Associated factors of poor knowledge about epilepsy, 2019.

Variable		Knowledge		COR (95% CI)	AOR (95% CI)
		Good	Poor		
Age	18–35	234	138	1	1
	35–45	67	63	1.59 (1.06, 2.39)^*∗*^	0.95 (0.58, 1.57)
	>45	35	59	2.86 (1.79, 4.56)^*∗*^	1.37 (0.74, 2.53)

Income	<3000	37	46	1	1
	≥3000	299	214	0.58 (0.36, 0.92)^*∗*^	0.78 (0.45, 1.37)

Occupation	Farmer or housewife	30	68	4.47 (2.66, 7.51)^*∗*^	1.39 (0 .69, 2.79)
	Private workers	95	75	1.56 (1.02, 2.37)^*∗*^	1.07 (0.64, 1.79)
	Daily laborer	34	27	1.56 (0.87, 2.80)	0.77 (0.36, 1.64)
	Student	43	22	1.01 (0.56, 1.82)	0.92 (0.41, 2.06)
	Gov't employee	134	68	1	1

Educational status	Lack of modern education	32	84	6.92 (4.21, 11.36)^*∗*^	3.74 (1.80, 7.70)^*∗∗*^
	1–8	52	43	2.18 (1.33, 3.57)^*∗*^	1.45 (0.78, 2.69)
	9–12	78	67	2.26 (1.47, 3.49)^*∗*^	2.13 (1.27, 3.59)
	>12	174	66	1	1

Marital status	Single	178	85	1	1
	Married	158	175	2.32 (1.66, 3.24)^*∗*^	1.88 (1.20, 3.21)^*∗*^

Family role	Family head	192	194	1	1
	Son or daughter	57	34	0.59 (0.37, 0.94)^*∗*^	1.45 (0.67, 3.15)
	Living alone	87	32	0.36 (0.23, 0.57)^*∗*^	0.70 (0.37, 1.32)

Witnessed a seizure	Yes	291	184	1	1
	No	45	76	2.67 (1.77, 4.03)^*∗*^	1.79 (1.10, 2.90)^*∗*^

Heard epilepsy	Yes	322	224	1	1
	No	14	36	3.70 (1.95, 7.01)^*∗∗*^	2.67 (1.27, 5.59)^*∗*^

^*∗*^ *p* value <0.05, ^*∗∗*^*p* value ≤0.001, COR = crude odds ratio, and AOR = adjusted odds ratio.

**Table 4:** Associated factors of at unfavorable attitude towards people living with epilepsy, 2019.

Variable		Attitude		COR (95% CI)	AOR (95% CI)
		Favorable	Unfavorable		
Sex	Male	221	121	1	1
	Female	139	125	1.56 (1.12, 2.17)^*∗*^	0.80 (0.59, 1.34)

Age	18–35	237	135	1	1
	35–45	67	63	1.65 (1.10, 2.47)^*∗*^	0.97 (0.57, 1.64)
	>45	46	48	1.83 (1.16, 2.89)^*∗*^	0.88 (0.46, 1.68)

Income	<1000	33	50	3.24 (1.94, 5.4)^*∗*^	2.52 (1.34, 4.70)^*∗*^
	1000–2999	133	110	1.77 (1.2, 2.5)^*∗*^	1.40 (0.90, 2.14)
	>3000	184	86	1	1

Educational status	Lack of modern education	40	76	5.57 (3.44, 9.01)^*∗*^	2.09 (1.1, 4.44)^*∗*^
	1–8	51	44	2.53 (1.54, 4.16)^*∗*^	1.26 (0.66, 2.41)
	9–12	80	65	2.38 (1.59, 3.69)^*∗*^	1.74 (0.90, 3.00)
	Grade 12 and above	179	61	1	1

Marital status	Single	180	83	2.07 (1.4, 2.91)^*∗*^	1.39 (0.80, 2.41)
	Married	170	163	1	1

Family role	Family head	201	185	1	1
	Son or daughter	60	31	0.52 (0.31, 0.86)^*∗*^	0.58 (0.25, 1.38)
	Living alone	89	30	0.39 (0.25, 0.61)^*∗*^	0.43 (0.22, 0.83)

Occupation	Farmer and housewife	31	67	5.23 (3.10, 8.83)^*∗*^	2.04 (0.97, 4.28)
	Private worker	101	69	1.65 (1.07, 2.54)	1.28 (0.74, 2.20)
	Daily laborer	33	28	2.05 (1.14, 3.70)^*∗*^	0.96 (0.43, 2.13)
	Student	42	23	1.32 (0.73, 2.39)	1.80 (0.75, 4.30)
	Government	143	59	1	1

Witnessed seizure	Yes	311	164	1	1
	No	39	82	3.98 (2.6, 6.1)^*∗*^	2.27 (1.35, 3.80)^*∗*^

Family with epilepsy	Yes	34	12	1	1
	No	316	234	2.09 (1.06, 4.13)^*∗*^	1.53 (0.73, 3.2)

Friend with epilepsy	Yes	105	46	1	1
	No	245	200	1.86 (1.25, 2.76)^*∗*^	1.24 (0.73, 2.20)

Heard the term epilepsy	Yes	341	205	1	1
	No	9	41	7.57 (3.6, 15.9)^*∗*^	4.73 (2.04, 11.00)^*∗*^

Walking time	<30 min	297	186	1	1
	30–61 min	53	60	1.8 (1.19, 2.73)^*∗*^	1.77 (1.06, 2.79)^*∗*^

^*∗*^ *p* value <0.05, COR = crude odds ratio, and AOR = adjusted odds ratio.

## Data Availability

The datasets used and/or analyzed during the current study are available from the corresponding author on reasonable request.
